# Why do ambulance services have different non-transport rates? A national cross sectional study

**DOI:** 10.1371/journal.pone.0204508

**Published:** 2018-09-21

**Authors:** Alicia O’Cathain, Richard Jacques, Tony Stone, Janette Turner

**Affiliations:** School of Health and Related Research (ScHARR), University of Sheffield, Sheffield, United Kingdom; Monash University, AUSTRALIA

## Abstract

**Background:**

Some patients calling ambulance services (known as Emergency Medical Services internationally) are not transported to hospital. In England, national ambulance quality indicators show considerable variation in non-transport rates between the ten large regional ambulance services. The aim of this study was to explain variation between ambulance services in two types of non-transport: discharge at scene and telephone advice.

**Methods:**

Mixed model logistic regressions using one month of data (November 2014) from the Computer Aided Despatch systems of the ten large regional ambulance services in England.

**Results:**

41% (251 677/615 815) of patients calling ambulance services were not transported to hospital. Most were discharged at scene after attendance by an ambulance (29% n = 182 479) and a small percentage were given telephone advice (7% n = 40 679). Discharge at scene rates varied by patient-level factors e.g. they were higher for elderly patients, where the reason for calling was falls, and for patients attended by paramedics with extended skills. These patient-level factors did not explain variation between ambulance services. After adjustment for patient-level factors, the following ambulance service level factors explained variation in discharge at scene rates: proportion of patients attended by paramedics with extended skills (odds ratio 1.05 (95% CI 1.04, 1.07)), the perception of ambulance service staff that paramedics with extended skills were established and valued within the workforce (odds ratio 1.84 (1.45, 2.33), and the perception of ambulance service staff that senior management viewed non-transport as risky (odds ratio 0.78 (0.63, 0.98)). Variation in telephone advice rates could not be explained.

**Conclusions:**

Variation in discharge at scene rates was explained by differences in workforce configuration and managerial motivation, factors that are largely modifiable by ambulance services.

## Introduction

Not all patients calling ambulance services, or Emergency Medical Services as they are known as in some countries, are transported to a hospital. Non-transport can occur because patients refuse to go to hospital or because ambulance clinicians make decisions not to take patients to a hospital. In some countries refusal to travel is the main reason for non-transport (sometimes called non-conveyance),[1] but in other countries ambulance services have policies and guidelines to allow staff to make decisions about whether to transport patients to hospital.[2] Non-transport rates vary by country and research study.[2, 3] For example, a systematic review of falls in older people identified that these varied between 11% and 56%;[3] a recent systematic review that included studies from North America, Europe, Australia, Asia and Africa identified larger variation in non-transport rates for the general patient population of between 4% and 94%.[2]

There are different types of non-transport to hospital. In the United Kingdom (UK) there are currently three main types in use: telephone advice to self-manage or contact another service, given by clinicians based in the ambulance service; discharge at scene after a face-to-face contact with an ambulance crew where the crew offer treatment and advice to patients; or transport by ambulance to a service other than a hospital with an emergency department, such as a walk-in or urgent care centre. These types of non-transport to a hospital are also in use internationally. For example, telephone advice was offered to one in ten calls to the ambulance service in a region in Australia.[4]

Ambulance services make an important contribution to health care provision, and must be considered when attempting to understand the quality and safety of health care.[5] Non-transport to hospital has the potential to improve the quality of care for a large number of patients each year, although evidence of these benefits is lacking currently. Avoiding a trip to hospital could potentially deliver benefits for patients, emergency departments and ambulance services. For patients, treatment at the scene without transport to a hospital could be the most appropriate clinical response to patients’ needs, thereby reducing the inconvenience of attending an emergency department far from their home. For emergency departments, ambulance services dealing with patients safely and appropriately without transport to hospital could reduce demand for emergency departments and therefore demand for emergency hospital beds,[6] allowing emergency departments to reduce waiting times for patients who need the level of clinical care they provide. For ambulance services, concerns have been expressed within the media about ambulances needing to queue outside overcrowded emergency departments in England. Non-transport of patients has the potential to remove the delays caused by this queuing, allowing ambulances to respond more quickly to other calls.

### Variation in non-transport rates between ambulance services

As well as variation in non-transport rates between countries, there is variation between ambulance services within countries. In England, Emergency Medical Services are provided by 11 ambulance services, of which 10 regional services cover 99% of the population of 55 million people. The national policy is to promote non-conveyance (as it is called in England) as a way of offering care close to home.[7] Ambulance services are fulfilling this remit by treating a large proportion of patients at the scene and not transporting them to hospital. Rates of non-transport are monitored as indicators of the quality of ambulance services and are published monthly for each ambulance service.[8] At the end of 2016, the rate of calls ending in telephone advice varied between 5% and 17%, the rate of calls sent an ambulance but not transported to a hospital varied between 23% and 51%, and overall non-transport rates varied between 40% and 68% for the 10 large regional services.

Variation in practice within health care can raise concerns about quality. It is important to identify the causes of variation in health care and encourage actions to deal with it.[9] Some variation may be warranted due to patient need or preference, and some may be unwarranted.[10, 11] Unwarranted variation can be related to differences in evidence-based practice, preferences held by service providers, or supply of resource. For example, variation in non-transport rates between ambulance services caused by differences in the supply of paramedics with extended skills would be unwarranted. It is also the case that some variation may be modifiable by ambulance services, e.g. workforce configuration, and some variation may not, e.g. the types of problems patients call an ambulance for.

### Determinants of non-transport

A recent systematic review identified a range of patient characteristics associated with non-transport including age, gender, ethnic group, geography, reason for call and vital signs.[2] There was little consistency in the direction or size of effect of patient-level determinants between studies included in this review; for example, higher non-transport rates were associated with males in some studies and females in others. There was consistent evidence of higher rates in children and elderly people, and for some reasons for calling the service such as falls and diabetic hypoglycaemia. Factors influencing the decision-making process around transport were related to the patient, professional, healthcare system and availability of decision support tools. Patient influences included the physical health and desire for transport of patients, and healthcare system influences included access to general practitioners and other healthcare services to allow discharge at scene. Ambulance personnel with extra training or extended skills had higher non-transport rates. This latter finding was supported by another systematic review and meta-analysis identifying that paramedics with extended skills had higher non-transport rates than conventional paramedics, although concerns were expressed about whether all potential confounders were adjusted for within analyses of individual studies.[12]

The evidence base does not always distinguish between different types of non-transport to hospital and tends to focus largely on discharge at scene. Nor does it address factors affecting variation in non-transport rates between ambulance services and therefore the influence of ambulance service characteristics. Variation in non-transport rates between ambulance services may be explained by differences in the types of people calling each ambulance service. This would not cause concern because it reflects differences in case-mix between ambulance services. However, variation explained by differences in practices between ambulance services would be a cause for concern because patients with the same health need in different parts of the country would be receiving different health care. A national study of Variation in Ambulance Non-conveyance (the VAN study) focused on understanding why there was variation in non-transport rates between ambulance services in England.[13] This mixed methods study included a qualitative interview study of staff in ambulance services to identify factors affecting non-transport and a quantitative study using routine data from ambulance services to identify factors explaining variation between ambulance services. The quantitative component of this study is reported here, aiming to explain variation in different types of non-transport between the ten large regional ambulance services in England.

## Methods

### Setting and context

In England in the UK Emergency Medical Services are provided by 11 ambulance services within the National Health Service (NHS) to the population of 55 million people. Ten of these ambulance services deal with over 99% of emergency ambulance calls from the population of England. Most calls are from patients calling 999 to request an ambulance–approximately four in five calls–with the remaining calls being passed directly through from patients calling a telephone helpline for urgent care called ‘NHS 111’, or through direct referral from a health professional requesting an emergency ambulance. Calls are taken by non-clinical staff who use software to identify the priority of each call. The ten ambulance services use one of two different priority dispatch systems: Medical Priority Dispatch System or NHS Pathways. A small percentage of calls that are categorised as low priority are passed to clinicians within each ambulance service for secondary telephone triage. These clinicians, physically based in each ambulance service, use a decision support software to determine whether the patient needs an ambulance or can be offered telephone advice only. Telephone advice includes self-care advice or referral to a service such as primary care; an ambulance is not dispatched. For calls not sent for secondary telephone triage, an ambulance is dispatched and patients may be attended by paramedics, paramedics with extended skills, or emergency care technicians with basic emergency training. The ambulance crew assesses and treats patients at scene and can discharge people at scene, transport them to a hospital-based emergency department, or transport them to another health facility such as a walk-in centre. Decisions are made by ambulance crews in conjunction with patients and their families. Local protocols exist for making decisions relating to transport of patients with some health conditions. Sometimes non-transport occurs due to patient refusal to travel.

### Wider study

The research reported here was part of a sequential mixed methods study of a qualitative component followed by a quantitative component.[13] The quantitative component, a statistical analysis of one month of routine data from the ten ambulance services, is the focus on this paper. However, because the qualitative findings were used within the quantitative analysis reported here, a brief description of the qualitative research is given. Further details of this qualitative component are reported elsewhere.[13] A qualitative interview study was undertaken with five staff from each of the ten large ambulance services in England (totalling 49 interviews). The aim was to identify factors perceived to affect different types of non-transport specifically within each interviewee’s ambulance service. Three types of staff were purposively selected to represent different perspectives within each ambulance service. Two managers were selected to offer a strategic view of non-transport within their ambulance service e.g. workforce configuration and training. These included Operational and Medical Directors and clinicians managing teams providing non-transport. Two paramedics were selected to offer the perspectives of staff making decisions about non-transport for individual patients. The healthcare commissioner for each ambulance service was selected to offer an external view of how the ambulance service engaged with non-transport. In England, healthcare commissioners hold budgets to buy health services for their geographically-based population. Each ambulance service has a lead healthcare commissioner who negotiates contracts about how the ambulance service will provide health care, including non-transport. Ambulance service-level factors were derived from this qualitative research and then tested in the quantitative component of the study (see section on ‘ambulance service-level factors’ for details). The National Research Ethics Service Committee North West–Greater Manchester West (REC reference 14/NW/1388)–approved this study.

### Data on non-transport rates

Routine data was requested from the Computer Aided Despatch systems of each of the 10 large ambulance services in England. Data on all emergency calls that received a telephone or face-to-face response from the ambulance service was requested for the single month of November 2014. The requests for data were made in May 2015. The patient transport service, providing pre-planned non-emergency transport, was not included in the requested data set. This single month of data was expected to consist of around 540,000 calls to the ambulance service (based on published ambulance quality indicators for England in November 2014). Selection of a longer time period would have resulted in a very large dataset, with statistical tests identifying very small differences as statistically significant. Selection of a smaller time period such as a week would have raised concerns about the potential for some ambulance services to experience unusual events within that week as a cause of variation between ambulance services. Call outcomes were telephone advice only, discharge at scene, transport to hospital with an emergency department, and transport to an alternative service. All datasets were fully anonymised and deidentified by ambulance services prior to sending to the research team.

### Patient-level factors

Potential patient-level factors were identified from the literature and the qualitative interviews undertaken as part of the wider study. Routine data was not available for some of the factors identified, in particular patient desire for transport to hospital. Routine data on factors was identified from three sources (see Table 1). First, the ambulance service Computer Aided Despatch (CAD) systems. Second, data from the 2011 census for England. Variables identified from the literature and qualitative research as affecting non-transport were identified within the publically available census data. These variables were linked to the CAD data using the lower super output area (LSOA) of each call which was provided by ambulance services with the CAD data. LSOAs are the smallest geography for which some census data are available, with a mean population size of around 1600. The LSOA based census factors described the area from which the call was made to an ambulance service rather than characteristics of the individual patient. Third, each ambulance service holds routine data about the skill-mix of crews attending each incident. We requested that the highest skill-mix of any crew attending each incident be linked to the CAD data. Labels used to describe crew members differed by ambulance service so the research team standardised labels (see Table 1 for details).

**Table 1:** Description of factors tested in the regression.

Level of factor	Factor	Groups	Description, including justification for groups	Source of data	% Missing values
Patient	Patient age	0–2, 3–10, 11–20, 21–30, 31–40, 41–50, 51–60, 61–70, 71–80, 81–90, > 90	Age was grouped because age is sometimes given by a caller who guesses the patient age. 0–2 group was used because some ambulances services reported different non-transport policies for children under 2 years old.	CAD	7.4%
	Patient sex	Female, Male	Only two categories available	CAD	6.2%
	Time of call	Out of Hours, In Hours	Time of call was dichotomised into ‘In hours = 8am-6pm weekdays’ and ‘Out of hours = all other times’. It was grouped because qualitative interviews identified perceptions that the availability of services that facilitated non-transport was better during normal working hours for health services.	CAD	0%
	Source of call	999, 111	In England patients call 999 directly or are passed to 999 after calling the urgent care telephone service NHS 111	CAD	0%
	Type of caller	Patient Health practitioner	All calls from a patient, family, friend or bystander were labelled as ‘patient’. Community nurses and general practitioners can call for an emergency ambulance on behalf of patients and were labelled ‘health practitioner’	CAD	Variable missing for one ambulance service (11.7%)
	Reason for call	Falls, Abdominal Pain, Breathing difficulties, Cardiovascular, Fitting, Injury, Psychiatric, Sick or Unconscious, Other	There are many codes for reason for call and they differ by the two triage software systems used by ambulance services. A small working group of clinicians, experts within the research team, and a research paramedic met to develop common categories from the two triage software systems and identified specific codes where a large proportion were not-transported; all other reasons were classed as ‘other’	CAD	29.4%
	Assessment of urgency	Red 1 & 2 (emergency), Green 1 & 2 (urgent), Green 3 & 4 (low acuity)	Codes such as Red 1 and 2 were grouped together due to small numbers	CAD	1.1%
	Indices of Multiple Deprivation (IMD)	Quintiles Q5 (Least Deprived), Q4, Q3, Q2, Q1 (Most Deprived)	The IMD is the official measure of deprivation of small areas in England, ranking every small area from 1 (most deprived) to over 30,000 (least deprived). It is based on seven aspects of deprivation including income and employment. Quintiles were used because this is a common approach to using IMD in regressions. The variable describes the area from which the call was made.	Census	3.7%
	Urban-rural status	Urban, Rural	The Rural Urban Classification is an official statistic used in the census to distinguish rural and urban areas. There are 4 urban and 6 rural categories. The urban/rural dichotomy was used. Rural areas are outside settlements with more than 10,000 resident population. The variable describes the area from which the call was made.	Census	3.7%
	% population with no central heating	Quintiles	The percentage of the population in the small area from which the call was made that reported having no central heating in the census. Used to represent the quality of housing of a patient.	Census	3.7%
	% population living alone	Quintiles	The percentage of the population in the small area from which the call was made that reported living alone in the census. Used to represent informal support unavailable for patients.	Census	3.7%
	% population with English not as their first language	Quintiles	The percentage of the population in the small area from which the call was made that reported not having English as their first language in the census. Used to represent ethnic groups where communication might affect decision to transport.	Census	3.7%
	% population with severe long term illness	Quintiles	The percentage of the population in the small area from which the call was made that reported having a severe long term illness. Used to represent health status of patients.	Census	3.7%
	Skill-mix	Paramedic, Paramedic extended skills, Other	Each ambulance service uses different labels and codes for the skill-mix of crew attending the scene. A small working group of clinicians, experts within the research team, and a research paramedic met to develop common categories for skill-mix. Each ambulance service was requested to link the labels and codes they used to these common categories, based on the highest skill-mix of attending ambulance crew. This data was not available in CAD but held in another routine dataset by ambulance services and was linked to the CAD data by the ambulance service. Paramedics with extended skills, or advanced paramedics as they are called in England, are defined by the national College of Paramedics as experienced autonomous paramedics with masters degrees in a subject relevant to their practice. ‘Other’ mainly included emergency medical technicians and a small number of doctors and nurses.	Ambulance routine data	Variable missing for one ambulance service and some missing values for all other services (14.4%)
Ambulance service	Workforce configuration	% patients attended by paramedics with extended skills	The patient-level variable on skill-mix was used to create an ambulance service-level variable of the percentage of calls attended by paramedics with extended skills to represent the size of the workforce made up of paramedics with extended skills within each ambulance service	Ambulance routine data	Missing for one service (14.4%)
	Complexity of emergency and urgent care system	Medium, high, low	In the qualitative interviews interviewees described how ambulance crews having to move between areas run by different healthcare commissioners (these are called clinical commissioning groups) reduced the ability to discharge at scene because each area had different services with different referral pathways which ambulance crew needed to know about in order to discharge at scene. Also, although a lead healthcare commissioner worked with the ambulance service to devise a contract for providing non-transport, sometimes individual commissioners from these clinical commissioning groups set up their own contracts with the ambulance service. The CAD system identified the number of clinical commissioning groups covered by each ambulance service to represent the complexity of the external system that an ambulance service had to deal with.	CAD	0%
	Type of triage software	AMPDS NHS Pathways	One ambulance service used two types of software in different geographical regions and was coded as using the software triaging the majority of callers	Ambulance Information Team	0%
	Stability of the organisation	No changes, Significant changes	Staff perceptions of changes occurring to senior management or the effects of external assessments of service quality	Qualitative study	0%
	Organisational motivation for non-transport	No view or mixed views, viewed as opportunity, risk aversion	Staff perceptions of motivation of senior management to undertake non-transport	Qualitative study	O%
	How extended paramedics are used	No view or mixed views, in limited capacity, established and valued	Staff perceptions of whether paramedics with extended skills were used in the ambulance service	Qualitative study	0%
	Fear of retribution	No evidence, Low levels of fear, Evidence of fear	Staff perceptions of level of fear of retribution amongst paramedics if non-transport resulted in adverse events	Qualitative study	0%
	Provision of services in the wider system	Inconsistent views, lacking in provision, good provision	Staff perceptions of availability of services in the wider emergency and urgent care system that facilitated non-transport	Qualitative study	0%
	Connectivity with wider system	Inconsistent views, lacking connectivity, good connectivity	Staff perceptions of how connected an ambulance service was to other services within the wider emergency and urgent care system	Qualitative study	0%
	Commissioners	Worked with some localities only, poor, good	Staff perceptions of the quality of the relationship between the ambulance service and their health care commissioners	Qualitative study	0%
	Telephone advice	Limited use, negative views, enthusiastic senior management	Staff perceptions of provision of this type of non-transport within their ambulance service. Applicable to telephone advice analysis only	Qualitative study	0%
	Cost per call	Medium, low, high	Cost per call was calculated by the National Audit Office by dividing an ambulance service’s urgent and emergency care income by the number of calls presented to its switchboard. It was calculated to represent cost-effectiveness. It was tested in the regression because it was available and varied by ambulance service rather than there being a clear rationale for its potential influence on non-transport. 10 ambulance services grouped into three groups of high, medium and low	National Audit Office	0%
	Cost per face-to-face attendance	Medium, low, high	See explanation for ‘cost per call’. 10 ambulance services grouped into three groups of high, medium and low	National Audit Office	0%
	Staff absence rate	Medium, low	10 ambulance services grouped into three groups of high, medium and low. No service had a higher rate so only two categories were used	National Audit Office	0%
	% frontline staff with extended skills	Medium, high	The percentage of frontline staff with extended skills is similar to the workforce configuration variable above. However, it also includes staff offering telephone advice and does not measure the percentage of incidents attended by different skill-mix. 10 ambulance services grouped into three groups of high, medium and low	National Audit Office	0%
	Income per head of population	Medium, low, high	10 ambulance services grouped into three groups of high, medium and low	National Audit Office	0%

### Ambulance service-level factors

Data on ambulance-service level factors were identified from three sources (see Table 1). First, three variables were identified from each ambulance service: from the CAD system, routine data held on skill-mix of crews attending incidents, and from the information team at each service (see Table 1). Second, eight variables were identified from the qualitative interview study in the wider mixed methods study in the following way. Interviews were transcribed verbatim. Transcripts were read to identify factors perceived to affect non-transport within that ambulance service. Each transcript was coded using this set of factors. For each factor, all relevant transcript excerpts from interviewees within an individual ambulance service was read and a summary statement about the factor for that ambulance service was produced. This was repeated for each factor and each ambulance service. Each factor was categorised as one of three levels for each ambulance service–positive/high, no views/mixed views, or negative/low. The middle category was used where there was conflicting information from different interviewees within an ambulance service about a specific factor, or where there was not enough information to make a judgement. This process is called ‘quantitizing’ [14] and was undertaken by a single researcher who was blind to the non-transport rates of each ambulance service. A matrix was produced with ambulance services as columns and factors as rows, with cells categorised as 1,2 or 3 [13]. This matrix was passed to the statistician for inclusion in the regression (see Table 1). Third, advantage was taken of a published national audit of ambulance services in England, where tables of ambulance characteristics were presented. [15] Five variables were identified from this National Audit Office report where there was variation between ambulance services in factors potentially important to non-transport (see Table 1). Variables were categorised as high, medium and low by a researcher.

### Analysis

Ambulance services linked their CAD data and their routine data on skill-mix. They included LSOA in the dataset and sent the data to the research team. The research team made efforts to reduce bias in these datasets by examining the data from each service in detail and interacting with information staff in each ambulance service to understand how CAD variables were coded and calculated. The research team used LSOA to link census data to the CAD data. Although the census variables were at small-area level rather than patient level, they were treated as patient level in the regression. The ambulance service-level variables (from the qualitative interviews, National Audit Office and routine ambulance data) were added to this dataset to allow a multi-level analysis to be undertaken on patient and ambulance-level variables. Specific hypotheses were not tested due to the variation in direction of effect of factors identified in a recent systematic review.[2]

Mixed effects logistic regression models with ambulance service as a random intercept were fitted in the statistical software R using the lme4 library.[16] Regressions were undertaken separately for calls ending in discharge at scene and calls ending in telephone advice. No analysis was undertaken on calls ending in transport to facilities other than hospitals with emergency departments because these included such a mix of acuities: low-acuity calls taken to minor injury units, end-of-life calls taken to hospices, and high acuity calls taken to specialist tertiary care e.g. patients with suspected stroke to a hyper-acute stroke unit. The denominator for the discharge at scene analysis was all calls sent an ambulance. The denominator for the telephone advice analysis was all calls.

There were two stages to the analysis. The first stage was to investigate which patient-level variables were statistically associated with non-transport. This was undertaken for discharge at scene because this accounted for the majority of non-transport. All patient-level variables listed in Table 1 were tested in the regression with the exception of one variable. Type of caller (patient or health practitioner) was removed from the analysis because there was no data available on this variable for one ambulance service and no healthcare professional calls recorded for another. The skill-mix variable was also missing for one of the ambulance services but, as this was identified in the evidence base as an important factor in non-transport, the analysis was conducted on the 9 ambulance services that provided skill-mix (the primary analysis) and then the final models were refitted on data from all 10 ambulance services with the skill-mix variable excluded (sensitivity analysis). Using a likelihood ratio test, all single patient-level variables that were significantly related to the outcome at p<0.1 were entered into the multivariable model building analysis. A backward elimination strategy with variables removed at P>0.05 was used to identify the subset of variables that independently predicted the outcome. As a final check, all variables excluded in the first stage of model selection were added to the model to see if they became important in the presence of others. First order interactions between predictor variables were investigated but, due to the large sample size, small effects were statistically significant and made the models difficult to interpret. Because of this all interactions were removed from the models. The patient-level variables identified at this stage were used to adjust for case-mix when testing ambulance service-level variables in both regressions (for discharge at scene and for telephone advice).

The second stage of the analysis was to consider ambulance service-level variables. All ambulance service-level variables listed in Table 1 were tested in the regression. Each ambulance service-level variable was added to the model with patient-level variables. All ambulance service-level variables from either the model with case-mix included or excluded with p<0.1 were entered into this stage of the analysis. All of these variables were added to the model and backward elimination was used to removed ambulance service-level variables with P>0.05.

Variability between ambulance services was assessed visually using caterpillar plots. The odds ratios of the random intercepts (ambulance service) were plotted for (i) the null model or intercept only model (i.e. the model with no patient or ambulance service-level variables); (ii) the model with only patient-level variables; and (iii) the model with both levels of variables. By comparing these plots it was possible to see how variability between random intercepts (ambulance services) changed as different levels of variables were added.

The approach used to investigate variation in discharge at scene rates was repeated for telephone advice only rates. For this analysis, urgency level, skill-mix and source of call were excluded because they were not relevant to these types of calls or data on these variables were not available: most telephone advice only calls are categorised as low acuity, there was little or no skill-mix data available, and national policy at November 2014 was that calls from a particular source (NHS 111) should not be re-triaged by clinicians in ambulance services so only calls sourced from 999 were included in the dataset.

## Results

### Description of types of non-transport

The dataset of 615 815 calls was larger than the one reported in national ambulance quality indicators for non-transport (538,865) in November 2014 because it included calls from NHS 111 which are excluded from the denominator of the national ambulance quality indicators. 41% (251 677/615 815) of patients calling ambulance services were not transported to hospital. Most non-transported patients were discharged at scene after attendance by an ambulance (29% n = 182 479 of all calls). Discharge at scene rates varied between 21% and 46% by ambulance service. A small percentage were given telephone advice only (7% n = 40 679 of all calls) or transported to an alternative service (4% n = 28 519 of all calls). Telephone advice only rates varied between 3% and 11% by ambulance service.

### Denominators and missing data

The reason for calling an ambulance was missing for a large proportion of calls (Table 1). Consideration was given to not including this variable in the analysis but it is a strong and consistent predictor of non-transport so it was included. For the discharge at scene analysis, the denominator was all patients with face to face contact with an ambulance crew including discharged at scene, transported to an emergency department and transported to an alternative service. A complete case analysis was undertaken on 343 875 patients of the 546 916 patients in the nine ambulance services with skill-mix data. For the sensitivity analysis on all ten ambulance services the complete case analysis was based on 370 656 of the 575 136 calls. For telephone advice, the denominator was all calls. A complete case analysis was undertaken on 400 630 of the 615 815 calls.

### Determinants of discharge at scene rates

**Fig 1:**
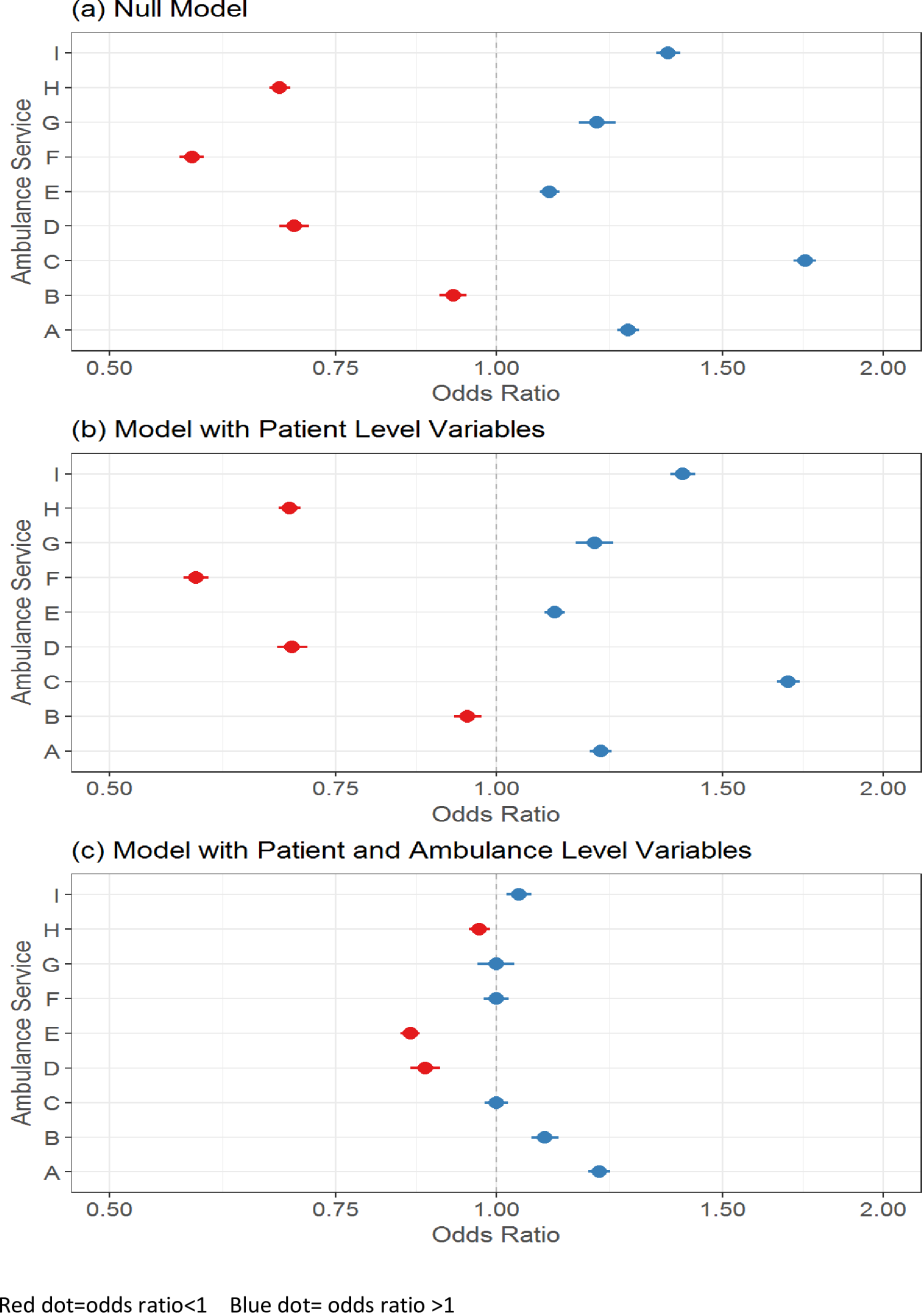
Variation in discharge at scene rates between ambulances services (based on 9 ambulance services with skill-mix data).

Only nine ambulance services were included in the ‘discharge at scene’ analysis because a key variable ‘skill-mix’ was not available for one ambulance service (see Table 1). There was considerable variation in discharge at scene rates between the nine ambulance services which provided data on skill-mix (see Fig 1A). When patient-level variables were tested, rates varied by age group (lower for children aged below two and higher for elderly patients), were slightly higher for men, lower in hours (8am-6pm weekdays), lower for any reason for calling that was not about falls, higher for calls classified as low urgency, higher for calls sourced from NHS 111, higher for calls attended by paramedics with extended skills, and higher for calls made from areas of social deprivation (see Table 2). Although different patient-level variables were associated with variation in discharge at scene rates, they did not explain the variation between ambulance services (see Fig 1B). Patient-level variables in the final model were age group, sex, time of call, source of call. reason for call, assessment of urgency, % population with severe long term illness, indices of multiple deprivation and skill-mix (see Table 2). When ambulance service-level variables were tested, variation in discharge at scene rates reduced considerably (see Fig 1C). Variation between ambulance services was explained by three ambulance service-level variables after adjustment for patient-level variables: discharge at scene rates were higher for ambulance services with higher proportions of calls attended by paramedics with extended skills, and ambulance services where staff described paramedics with extended skills as an established and valued part of the workforce; rates were lower for ambulance services where staff described the management as risk averse to non-transport (see Table 2 for variables included in final model). A sensitivity analysis was undertaken by excluding skill-mix variables and thereby including all 10 ambulance services. This regression failed to explain variation between ambulance services, although the odds ratios of both patient-level and ambulance service-level variables were very similar to those in the regression that included skill-mix (final model shown in Table 2).

**Table 2:** Factors explaining variation in discharge at scene rates.

Variable	9 ambulance services (with skill-mix data)					10 ambulance services (no skill-mix data)		
Patient-level	No of calls in denominator	No of calls resulting in discharge at scene	Unadjusted Odds Ratio+ (95% CI)	Adjusted Odds Ratio (95% CI)	P-Value	Unadjusted Odds Ratio+ (95% CI)	Adjusted Odds Ratio (95% CI)	P-Value
Age group								
0–2	13,116	2,982	1	1	-	1	1	-
3–10	9,204	2,523	1.30 (1.22, 1.37)	1.35 (1.27, 1.44)	<0.001	1.29 (1.22, 1.37)	1.35 (1.27, 1.43)	<0.001
11–20	24,133	7,764	1.60 (1.53, 1.68)	1.66 (1.58, 1.75)	<0.001	1.60 (1.53, 1.67)	1.66 (1.58, 1.74)	<0.001
21–30	36,413	12,453	1.82 (1.75, 1.90)	1.91 (1.82, 2.00)	<0.001	1.81 (1.73, 1.88)	1.89 (1.81, 1.98)	<0.001
31–40	29,271	9,345	1.66 (1.59, 1.73)	1.76 (1.68, 1.84)	<0.001	1.65 (1.58, 1.72)	1.75 (1.67, 1.83)	<0.001
41–50	31,789	9,722	1.53 (1.46, 1.59)	1.63 (1.55, 1.71)	<0.001	1.52 (1.46, 1.58)	1.62 (1.55, 1.70)	<0.001
51–60	30,517	9,214	1.49 (1.43, 1.56)	1.60 (1.52, 1.68)	<0.001	1.47 (1.41, 1.54)	1.58 (1.51, 1.66)	<0.001
61–70	35,076	10,632	1.46 (1.40, 1.53)	1.52 (1.45, 1.60)	<0.001	1.44 (1.38, 1.50)	1.50 (1.44, 1.58)	<0.001
71–80	50,162	16,039	1.57 (1.51, 1.63)	1.54 (1.47, 1.62)	<0.001	1.53 (1.47, 1.60)	1.52 (1.45, 1.59)	<0.001
81–90	61,972	22,920	1.91 (1.84, 1.99)	1.75 (1.67, 1.83)	<0.001	1.88 (1.81, 1.95)	1.73 (1.66, 1.81)	<0.001
> 90	22,222	9,248	2.29 (2.19, 2.39)	1.99 (1.89, 2.09)	<0.001	2.25 (2.15, 2.35)	1.97 (1.87, 2.07)	<0.001
Sex								
Female	163,599	52,053	1	1	-	1	1	-
Male	180,276	60,789	1.08 (1.06, 1.10)	1.02 (1.00, 1.03)	0.034	1.07 (1.06, 1.09)	1.01 (1.00, 1.03)	0.064
Time of call								
Out of Hours	224,933	75,996	1	1	-	1	1	-
In Hours	118,942	36,846	0.87 (0.85, 0.88)	0.87 (0.85, 0.88)	<0.001	0.87 (0.85, 0.88)	0.87 (0.86, 0.88)	<0.001
Reason for call								
Falls	58,411	25,828	1	1	-	1	1	-
Abdominal Pain	8,321	2,046	0.36 (0.35, 0.38)	0.34 (0.32, 0.36)	<0.001	0.36 (0.35, 0.38)	0.34 (0.32, 0.36)	<0.001
Breathing difficulties	43,341	11,729	0.49 (0.48, 0.50)	0.62 (0.60, 0.63)	<0.001	0.49 (0.48, 0.51)	0.62 (0.60, 0.63)	<0.001
Cardiovascular	58,189	13,040	0.37 (0.36, 0.38)	0.46 (0.45, 0.48)	<0.001	0.37 (0.36, 0.38)	0.46 (0.45, 0.47)	<0.001
Fitting	16,625	4,766	0.53 (0.51, 0.55)	0.64 (0.62, 0.67)	<0.001	0.54 (0.52, 0.56)	0.65 (0.62, 0.67)	<0.001
Injury	44,616	14,099	0.53 (0.52, 0.55)	0.55 (0.54, 0.57)	<0.001	0.54 (0.53, 0.55)	0.56 (0.55, 0.58)	<0.001
Psychiatric	9,227	3,293	0.74 (0.70, 0.77)	0.74 (0.71, 0.78)	<0.001	0.74 (0.71, 0.77)	0.74 (0.70, 0.77)	<0.001
Sick or Unconscious	52,478	17,063	0.63 (0.61, 0.64)	0.71 (0.69, 0.72)	<0.001	0.63 (0.61, 0.64)	0.70 (0.68, 0.72)	<0.001
Other	52,667	20,978	0.70 (0.69, 0.72)	0.77 (0.75, 0.80)	<0.001	0.67 (0.66, 0.69)	0.74 (0.73, 0.76)	<0.001
Urgency Level								
Red 1 & 2 (emergency)	167,581	45,759	1	1	-	1	1	-
Green 1 & 2 (urgent)	139,292	50,417	1.45 (1.43, 1.48)	1.25 (1.23, 1.27)	<0.001	1.44 (1.42, 1.47)	1.24 (1.22, 1.26)	<0.001
Green 3 & 4 (low acuity)	37,002	16,666	2.25 (2.20, 2.30)	1.90 (1.86, 1.95)	<0.001	2.21(2.16, 2.26)	1.87 (1.82, 1.91)	<0.001
Source of call								
999	336,915	109,995	1	1	-	1	1	-
NHS 111	6,960	2,847	1.33 (1.27, 1.40)	1.46 (1.38, 1.53)	<0.001	1.35 (1.29, 1.41)	1.43 (1.37, 1.50)	<0.001
Skill-mix								
Paramedic	278,804	88,851	1	1	-	-	-	-
Paramedic with extended skills	23,189	10,111	1.36 (1.33, 1.40)	1.38 (1.34, 1.42)	<0.001	-	-	-
Other	41,882	13,880	1.00 (0.98, 1.03)	0.96 (0.94, 0.99)	0.002	-	-	-
% severe long term illness								
Q1 (Lowest)	68,123	25,529	1	1	-	1	1	-
Q2	70,079	22,553	0.96 (0.94, 0.98)	0.95 (0.93, 0.97)	<0.001	0.96 (0.94, 0.99)	0.95 (0.93, 0.98)	<0.001
Q3	70,302	23,443	1.01 (0.94, 0.98)	0.99 (0.96, 1.01)	0.301	1.00 (0.98, 1.02)	0.98 (0.96, 1.01)	0.162
Q4	70,225	23,334	1.00 (0.98, 1.03)	0.97 (0.95, 1.00)	0.039	1.00 (0.98, 1.03)	0.97 (0.95, 1.00)	0.048
Q5 (Highest)	65,146	20,983	1.05 (1.02, 1.08)	1.01 (0.98, 1.04)	0.368	1.04 (1.03, 1.07)	1.00 (0.98, 1.03)	0.798
Indices of Multiple Deprivation								
Q5 (Least Deprived)	44,140	15,378	1	1	-	1	1	-
Q4	47,526	18,953	1.00 (0.98, 1.03)	1.02 (0.99, 1.04)	0.265	1.01 (0.99, 1.04)	1.02 (0.99, 1.05)	0.111
Q3	46,859	22,807	1.03 (1.00, 1.05)	1.04 (1.02, 1.07)	0.002	1.03 (1.01, 1.06)	1.05 (1.03, 1.08)	<0.001
Q2	46,891	25,994	1.01 (0.99, 1.04)	1.05 (1.02, 1.08)	0.001	1.02 (1.00, 1.04)	1.05 (1.03, 1.08)	<0.001
Q1 (Most Deprived)	44,163	29,710	0.99 (0.97, 1.02)	1.05 (1.02, 1.08)	0.002	1.00 (0.98, 1.03)	1.05 (1.02, 1.08)	<0.001
**Ambulance service-level**								
% calls attended by paramedics with extended skills	343,875	112,842	1.04 (1.02, 1.08)	1.05 (1.04, 1.07)	<0.001	-	-	-
Organisational Motivation								
No view or mixed views	260,597	85,540	1	1	-	1	1	-
Opportunity	36,625	16,925	1.74 (1.36, 2.23)	0.80 (0.58, 1.11)	0.176	1.88 (1.14, 3.09)	1.38 (0.64, 2.97)	0.407
Risk Aversion	46,653	10,377	0.58 (0.47, 0.72)	0.78 (0.61, 0.98)	0.036	0.63 (0.42, 0.93)	0.70 (0.39, 1.29)	0.255
How extended paramedics are used								
No view or mixed views	138,210	40,360	1	1	-	1	1	-
In limited capacity	153,896	49,926	1.16 (0.96, 1.41)	1.06 (0.91, 1.24)	0.460	1.29 (1.11, 1.49)	1.19 (0.75, 1.87)	0.460
Established and valued	51,769	22,556	1.71 (1.43, 2.05)	1.82 (1.31, 2.34)	<0.001	1.95 (1.57, 2.42)	1.52 (0.81, 2.84)	0.191

+ these are odds ratios from a univariable logistic random effects model with ambulance service as a random intercept rather than a true unadjusted value (i.e. from a logistic regression model with no random effect)

### Determinants of telephone advice rates

**Fig 2:**
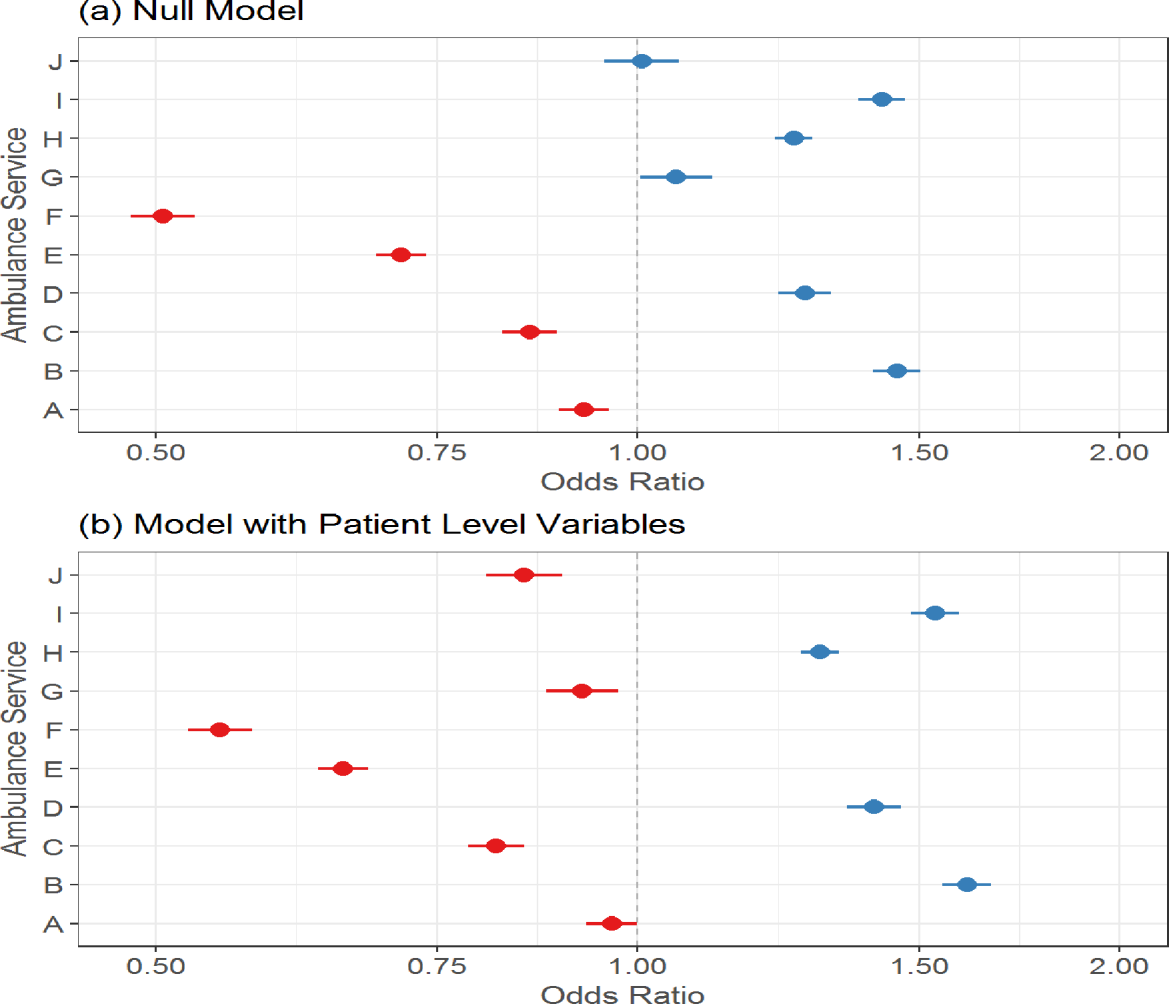
Variation in telephone advice rates between ambulances services (based on 10 ambulance services).

There was no skill-mix data available for telephone advice only calls so the primary analysis was undertaken on all 10 ambulance services. There was considerable variation between ambulance services for their telephone advice rates (see Fig 2A). When patient-level variables were tested, rates were lower for older age groups, slightly higher for men, lower for calls made in-hours and higher where the reason for the call was abdominal pain (see Table 3 for patient-level variables in final model). Variation between ambulance services was not explained by patient-level variables (see Fig 2B). No ambulance service-level variables were statistically significant. Therefore variation in telephone advice rates between ambulance services could not be explained by the variables tested here.

**Table 3:** Factors explaining variation in telephone advice rates.

	Calls	Telephone	Unadjusted	Adjusted	P-Value
	N	Advice	Odds Ratio+	Odds Ratio	
		N	(95% CI)	(95% CI)	
**Patient-level**					
Age group					
0–2	15,463	1,448	1	1	-
3–10	11,134	1,228	1.20 (1.12, 1.29)	1.22 (1.12, 1.32)	<0.001
11–20	28,778	2,629	0.98 (0.92, 1.03)	0.81 (0.75, 0.86)	<0.001
21–30	43,886	4,678	1.15 (1.09, 1.22)	0.93 (0.87, 0.99)	0.019
31–40	34,790	3,461	1.07 (1.01, 1.13)	0.91 (0.85, 0.97)	0.004
41–50	37,357	3,006	0.85 (0.80, 0.90)	0.78 (0.73, 0.84)	<0.001
51–60	35,657	2,646	0.78 (0.74, 0.82)	0.77 (0.72, 0.82)	<0.001
61–70	40,604	2,686	0.69 (0.65, 0.73)	0.70 (0.65, 0.75)	<0.001
71–80	57,545	3,307	0.59 (0.56, 0.62)	0.70 (0.56, 0.64)	<0.001
81–90	70,687	3,814	0.55 (0.52, 0.58)	0.61 (0.53, 0.60)	<0.001
>90	24,729	1,071	0.43 (0.40, 0.46)	0.45 (0.41, 0.49)	<0.001
Sex					
Female	190,346	14,205	1	1	-
Male	210,284	15,769	1.00 (0.98, 1.03)	1.02 (1.00, 1.05)	0.100
Time of call					
Out of Hours	263,370	21,290	1	1	-
In Hours	137,260	8,684	0.76 (0.74, 0.78)	0.81 (0.79, 0.83)	<0.001
Reason for call					
Falls	62,881	2,595	1	1	-
Abdominal Pain	11,950	3,098	9.10 (8.61, 9.61)	7.36 (6.94, 7.80)	<0.001
Breathing difficulties	45,972	1,578	0.83 (0.78, 0.88)	0.69 (0.64, 0.73)	<0.001
Cardiovascular	61,421	1,614	0.62 (0.59, 0.66)	0.55 (0.52, 0.59)	<0.001
Fitting	17,560	469	0.64 (0.58, 0.70)	0.48 (0.43, 0.53)	<0.001
Injury	54,291	4,961	2.32 (2.22, 2.43)	1.89 (1.80, 1.99)	<0.001
Psychiatric	10,600	1,005	2.46 (2.29, 2.64)	1.86 (1.72, 2.02)	<0.001
Sick or Unconscious	61,557	7,806	3.37 (3.22, 3.52)	2.93 (2.79, 3.07)	<0.001
Other	74,398	6,848	2.69 (2.57, 2.82)	2.18 (2.07, 2.29)	<0.001
% Severe Long Term illness					
Q1 (Lowest)	79,009	6,803	1	1	-
Q2	80,039	6,289	0.94 (0.91, 0.97)	0.97 (0.94, 1.01)	0.176
Q3	80,673	6,029	0.92 (0.89, 0.96)	0.96 (0.92, 1.00)	0.034
Q4	81,359	5,839	0.93 (0.90, 0.96)	0.96 (0.92, 1.01)	0.093
Q5 (Highest)	79,550	5,014	0.85 (0.81, 0.89)	0.89 (0.85, 0.93)	<0.001
Indices of Multiple Deprivation					
Q5 (Least Deprived)	51,250	3,637	1	1	-
Q4	63,422	4,568	1.03 (0.99, 1.08)	1.02 (0.97, 1.07)	0.493
Q3	76,211	5,536	1.05 (1.01, 1.10)	1.01 (0.96, 1.06)	0.692
Q2	93,931	7,163	1.11 (1.06, 1.15)	1.01 (0.96, 1.06)	0.714
Q1 (Most Deprived)	115,816	9,070	1.24 (1.19, 1.29)	1.11 (1.06, 1.17)	<0.001

## Discussion

### Summary of findings

41% (251 677/615 815) of patients calling ambulance services were not transported to hospital. Most non-transported patients were discharged at scene after attendance by an ambulance (29% n = 182 479) and a small percentage were given telephone advice only (7% n = 40 679). Discharge at scene rates were higher for older patients, men, patients calling out of hours, where the reason for calling was falls, calls categorised as non-emergency, patients calling from areas of social deprivation, and patients attended by paramedics with extended skills. These patient-level factors did not explain variation between ambulance services. After adjustment for patient-level factors, three ambulance service-level factors explained variation in discharge at scene rates. Rates were higher for services with a higher proportion of patients attended by paramedics with extended skills, and where the perception of ambulance service staff was that paramedics with advanced skills were established and valued within the workforce. Rates were lower where the perception of ambulance service staff was that senior management viewed non-transport as risky. Variation in telephone advice rates between ambulance services could not be explained by the variables tested here.

### Context of other research

The patient-level factors explaining variation in non-transport rates in this study were similar to those found in a recent systematic review of non-transport: age, gender, and reason for call.[2] The higher rate of non-transport found in this study for paramedics with extended skills has been found in a recent systematic review, although the odds ratio of 1.4 was considerably lower than the pooled odds ratio of 10.5 found in the review.[12] However, a number of potential confounders were adjusted for in the study reported here, unlike in individual studies included in the systematic review.[12]

A large number of variables were tested but they did not explain variation in telephone advice rates. Telephone advice offered to patients calling for an emergency ambulance is under researched. A recent systematic review identified such a small number of studies that the conclusion was that it was difficult to generalise from the evidence base.[17]

### Strengths and limitations

This is one of the first studies attempting to explain variation in non-transport rates between different ambulance services. The dataset was large and a wide range of variables was tested. There were six limitations. First, although there is national guidance on the types of calls to categorise as non-transported, ambulance services have different CAD systems and different staff produced the data sets amalgamated for this study. It is possible that some variation between ambulance services was explained by these differences rather than actual differences in rates. The research team attempted to check for differences and standardise the data but it was not possible to remove all differences. Second, data was missing for some variables for some ambulance services and a complete case analysis was undertaken. This reduced the size of the dataset and limited the analysis to cases were characteristics were available. This is likely to have introduced bias but the size and direction of bias is difficult to determine given the limited understanding of the types of cases more likely to have missing data. Third, there is little consistency of language when describing skill-mix within ambulances services. We addressed this by sending each ambulance service a set of generic skill-mix labels to link with their skill-mix codes but there was still room for different interpretation of the skill-mix types by the different ambulance services. Fourth, some of the ambulance-level variables tested were derived from qualitative interviews with ambulance staff. Only five interviews were undertaken within each ambulance service so data saturation was not necessarily achieved, leaving concerns that only a partial view of non-transport within each ambulance service was obtained. It is also possible that knowledge of their ambulance service performance for non-transport rates relative to other ambulance services may have led some interviewees to describe their ambulance in a way that matched their rate. It is also possible that coding of qualitative interview data was influenced by the research team’s knowledge of the rates for each ambulance service even though researchers were blinded to this until the coding of qualitative analysis was complete. It is not possible to estimate the magnitude of any bias introduced by this but the regression is likely to overestimate the amount of variation explained by these factors. Fifth, some factors have been identified elsewhere as influencing paramedic decision-making in non-transport and were not tested here due to lack of availability of data e.g. pressures of demand for ambulance services, and the level of training paramedics receive.[18] Finally, the data was from November 2014 and is being published in 2018 due to unexpected delays between requesting the data and obtaining and checking datasets from each ambulance service. There were no significant changes to national policy or guidelines for non-transport in England between 2014 and the publication of this article. The only major change to the ambulance service in England related to response times rather than non-transport, giving services longer to make a decision about the level of resource to send to a call. This is unlikely to have affected the relevance of the findings of this study. Nonetheless, it is important to acknowledge that this analysis is based on 2014 data and that it is necessary to continue to measure and assess the variability of non-transport rates between different ambulance services. There may also be a limitation in terms of using only one month if use of ambulances services differs by time of year.

### Implications

Variation in health care is difficult to interpret unless the gold standard level is known. There is no consensus on the optimal non-transport rate for an ambulance service. It is easy to draw the conclusion that the services in England with the highest rates are best and that the other services must change their practices to increase rates. Establishing the optimal rate would require further modelling of the cost-effectiveness of non-transport options,[19] and the safety and appropriateness of different rates of non-transport. Some approaches to non-transport may not be cost-effective, may result in mortality or increased severity of illness, may increase health service costs through additional subsequent service contacts, or may simply delay conveyance to hospital. Evidence suggests that concerns around safety and appropriateness may be unfounded for both discharge at scene [2] and telephone advice,[17] although it is still unknown whether adverse event rates are dependent on rates of non-transport.[20] In a context where national policy promotes care closer to home,[7] some patients call the ambulance service when they could have contacted a GP,[21] patients can gain the reassurance they seek without being transported to hospital,[22] and where there is little evidence to suggest that non-transport is unsafe,[2, 17] this study identified ways in which ambulance services with lower rates of discharge at scene could increase those rates. Some of the unwarranted variation between ambulance services lay within the control of the ambulance service, determined by their workforce policies and motivation to undertake discharge at scene.

It is important to reduce variation in practices between ambulance services so that patients within the same country receive a similar service. There is a national policy drive currently in operation in England to improve ambulance services. Increasing non-transport rates so that care is offered close to the patient’s home is a key part of this national initiative. The focus of this policy includes standardising practices between ambulance services and skilling the workforce to increase non-transport rates safely. The findings of this study have been presented to the leaders of the national ambulance improvement initiative to help contribute to planned improvements. There is also national guidance under development for emergency and acute medical care in over 16s with a recommendation for more non-transport.[23]

The findings are based on one month’s data in 2014 for the 10 large ambulance services in England which is likely to be generalizable to other time periods because publicly recorded non-transport rates did not vary by month during the year of 2014.[8] The findings are likely to be transferable to England in future years because although non-transport rates increased over time, and definitions of ambulance quality indicators changed over time,[8] the ranking of ambulance services remained stable over time. However, it is important to continue to measure the variability of non-transport rates between ambulance services and determine the reasons continued variability. The results presented here could be used as a baseline for measuring change in variability. The transferability of findings to ambulance services outside England is dependent on how these services are provided within different countries, whether there is a statutory obligation to transport patients to hospital, the level of non-transport in operation, and the extent to which non-transport rates are based on ambulance crew decision-making or patient refusal to travel.

## Conclusions

The factors explaining variation in non-transport rates between ambulance services differ by type of non-transport. Variation in discharge at scene rates between ambulance services in England was found to be largely unwarranted. It was explained by differences in workforce configuration and managerial motivation, factors that are largely modifiable by ambulance services. Variation in telephone advice rates between ambulance services could not be explained by variables tested in this study.
